# The Role of Rehabilitation Program in Managing the Triad of Sarcopenia, Obesity, and Chronic Pain

**DOI:** 10.3390/life15081174

**Published:** 2025-07-24

**Authors:** Bianca Maria Vladutu, Daniela Matei, Amelia Genunche-Dumitrescu, Constantin Kamal, Magdalena Rodica Traistaru

**Affiliations:** 1 Doctoral School, University of Medicine and Pharmacy of Craiova, 200349 Craiova, Romania; biancamariavld@gmail.com; 2 Department of Medical Rehabilitation, University of Medicine and Pharmacy of Craiova, 200349 Craiova, Romania; daniela.matei@umfcv.ro (D.M.); rodica.traistaru@umfcv.ro (M.R.T.); 3 Department of Internal Medicine, University of Medicine and Pharmacy Craiova, 200349 Craiova, Romania; amelia_genunche@yahoo.com; 4 Department of Family Medicine, University of Medicine and Pharmacy of Craiova, 200349 Craiova, Romania

**Keywords:** sarcopenic obesity, chronic pain, rehabilitation, SarQoL, SPPB, pressure pain threshold, quality of life

## Abstract

Background: Sarcopenic obesity, characterized by reduced skeletal muscle mass and excess adiposity, is strongly associated with chronic pain and functional decline in older adults. Objective: This prospective controlled trial without randomization investigated the effects of a structured, three-phase rehabilitation program on physical performance, pain, and sarcopenia-specific quality of life in elderly patients with sarcopenic obesity and chronic pain. Methods: In this study, 82 participants were enrolled and allocated to a study group (SG, n = 40), receiving supervised rehabilitation, nutritional counseling, and supplementation, or to a control group (CG, n = 42), which did not receive rehabilitation. The final analysis included 35 patients in SG and 36 in CG. Outcomes were assessed at baseline and six months using the Sarcopenia Quality of Life Questionnaire (SarQoL), Short Physical Performance Battery (SPPB), Numeric Rating Scale (NRS), and Pressure Pain Threshold (PPT). Results: The SG showed significant improvements in all outcomes: SarQoL increased from 57.02 to 63.98, SPPB increased from 7.14 to 8.4, PPT increased from 69.31 to 78.05, and NRS decreased from 6.94 to 4.65 (all *p* < 0.001). The CG showed no significant changes. Conclusions: The implementation of a structured, three-phase rehabilitation program resulted in clinically and statistically significant improvements in physical performance, pain perception, and sarcopenia-related quality of life in older adults with sarcopenic obesity and chronic pain.

## 1. Introduction

The concurrent rise in global life expectancy and obesity rates has led to the emergence of a complex geriatric syndrome—sarcopenic obesity (SO)—characterized by the coexistence of excess adiposity and low muscle mass and function [1,2]. This dual burden is becoming increasingly relevant in the context of aging populations, affecting physical independence, increasing the risk of disability, and reducing quality of life [3,4]. Definitions of SO have evolved significantly, with the most recent consensus provided by the European Society for Clinical Nutrition and Metabolism (ESPEN) and the European Association for the Study of Obesity (EASO) in 2022, which described SO as a pathological condition combining sarcopenia [5] and obesity, diagnosed based on skeletal muscle mass, muscle function, and fat mass [6]. However, the lack of universally accepted diagnostic criteria still poses major challenges in estimating its prevalence, implementing therapeutic strategies, and conducting epidemiological studies [7,8].

The estimated prevalence of SO varies widely depending on the setting and definition used, ranging from 2.1% to 12% in earlier reports and up to 23% in recent clinical studies applying ESPEN/EASO criteria [9,10].

Several pathophysiological mechanisms contribute to SO, including systemic low-grade inflammation, insulin resistance, hormonal dysregulation, mitochondrial dysfunction, and oxidative stress [11,12]. These mechanisms are often exacerbated by age-related changes, sedentary behavior, and nutritional deficits, leading to both muscle catabolism and fat accumulation [13].

A particularly important but underexplored dimension in SO is the presence of chronic pain. Both sarcopenia and obesity are independently associated with chronic pain syndromes; however, studies investigating their combined effect on pain experience and functionality are scarce [14]. Obesity has been linked with multiple types of chronic pain—including musculoskeletal, neuropathic, and visceral pain—through mechanical overload, systemic inflammation, and altered nociceptive processing [15,16]. Similarly, sarcopenia has been associated with impaired mobility, frailty, and musculoskeletal discomfort, especially in the lower back and lower limbs, leading to reduced physical activity and functional decline [17,18]. Previous work by our team has shown that inflammation negatively impacts physical performance even in overweight children, suggesting an early link between excess weight, systemic inflammation, and functional decline [19]. These findings reinforce the relevance of addressing obesity-related functional impairments early in life and support the rationale for comprehensive interventions targeting the triad of obesity, sarcopenia, and chronic pain later in adulthood.

Contrary to a generic context, the coexistence of sarcopenia, obesity, and chronic pain represents a specific and complex clinical triad. Each of these conditions contributes to functional decline and frailty, but their interaction exacerbates disability, complicates management, and requires an integrated rehabilitation approach. Emerging evidence suggests a bidirectional relationship between chronic pain and the components of SO, where pain contributes to physical inactivity, muscle disuse, and further weight gain, while excess adiposity and sarcopenia exacerbate pain through biomechanical stress and inflammatory pathways [20,21]. Despite their intertwined pathophysiology, the impact of chronic pain in patients with SO remains poorly addressed in the literature. Moreover, chronic pain in older adults is often under-recognized and undertreated, leading to significant functional impairment and emotional distress [22,23].

Given this context, the evaluation of chronic pain in patients with SO is not only clinically relevant but essential for developing personalized, multidimensional rehabilitation strategies. Addressing chronic pain in these patients may significantly improve their engagement in physical therapy, functional recovery, and ultimately their quality of life [24,25].

Current rehabilitation strategies for SO largely rely on resistance and aerobic training, nutritional interventions, and adjunctive therapies. However, the integration of multimodal physiotherapeutic interventions—including electrotherapy, low-level laser therapy, and deep oscillation therapy—into comprehensive programs targeting SO patients with chronic pain has not been systematically evaluated. Most previous studies have focused either on sarcopenia or obesity alone, with limited data on their combined effect when chronic pain is also present.

To our knowledge, this is the first study to explore the interrelation between sarcopenic obesity, chronic pain, physical performance, and quality of life in older adults. Our primary objective was to investigate whether a structured, phased rehabilitation program could improve physical performance and health-related quality of life (HRQoL) in older adults with SO and chronic pain. Given the comprehensive nature of the intervention, it was not designed to isolate the individual contribution of each treatment modality. Instead, our aim was to reflect the real-life clinical scenario where multimodal therapies are typically applied in combination. Nonetheless, future studies using factorial designs are necessary to dissect the relative efficacy of each element. A secondary objective was to assess the medium-term effect of the rehabilitation program in reducing pain and improving functional capacity in this vulnerable population.

This prospective controlled trial without randomization aims to contribute to filling a significant gap in the literature by providing novel evidence on the functional and symptomatic outcomes of an integrated rehabilitation approach tailored to the complex clinical profile of patients with SO and chronic pain.

## 2. Materials and Methods

### 2.1. Design Overview

This prospective, controlled, observational study was conducted between January and December 2024 in the Department of Physical Medicine and Rehabilitation at Filan tropia Hospital, Craiova, Romania.

Based on an initial screening of 360 consecutive patients presenting with joint and musculoskeletal disorders and chronic pain, 145 individuals met the preliminary inclusion criteria for sarcopenia and obesity. Following further diagnostic evaluation using the ESPEN and EASO algorithm and European Working Group on Sarcopenia in Older People (EWGSOP) guidelines, a final cohort of 82 patients diagnosed with sarcopenic obesity and chronic pain was selected for study enrollment. The patients aged 65–80 years, diagnosed with SO and chronic musculoskeletal pain, were clinically, paraclinically, and functionally assessed at baseline (T1) by a multidisciplinary team. Based on their expressed willingness to participate in a structured rehabilitation program, patients were allocated to either of the following groups:

- Study group (SG): 40 participants engaged in a 6-month tailored rehabilitation program;
- Control group (CG): 42 participants maintained their usual daily routine.

A follow-up evaluation (T2) was conducted 6 months after baseline, assessing pain (Numeric Rating Scale [NRS], Pressure Pain Threshold [PPT]), physical performance (Short Physical Performance Battery [SPPB]), and quality of life (Sarcopenia Quality of Life Questionnaire [SarQoL]). Body composition metrics such as skeletal muscle mass index (SMMI) and body fat percentage (BF%) were also recorded.

**Figure 1:**
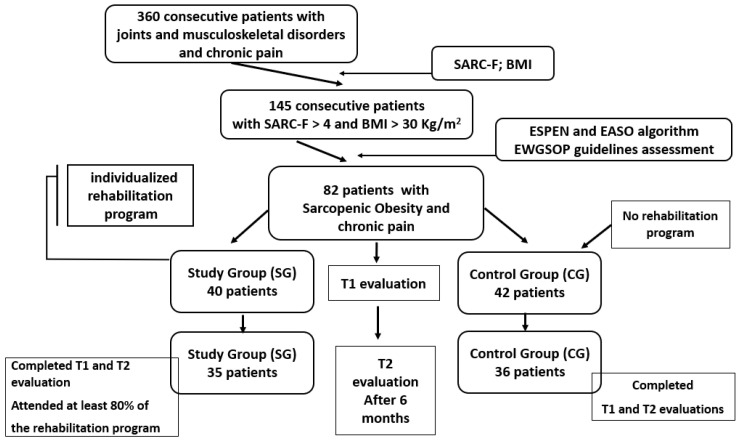
Diagram of study.

Only patients who completed both T1 and T2 evaluations, attended at least 80% of rehabilitation sessions, and had valid datasets were included in the final analysis. The reduction in participant numbers at the second evaluation was due to loss to follow-up and voluntary withdrawal. Specifically, 7 patients (3 in the study group and 4 in the control group) were lost to follow-up due to relocation or lack of contact, while 4 patients (2 in each group) chose to withdraw from this study for personal reasons. Figure 1 presents the study flow diagram.

### 2.2. Participants

#### 2.2.1. Eligibility Criteria

Inclusion criteria: (a) Chronic musculoskeletal pain persisting for more than 3 months; (b) mild to moderate osteoarthritis of the hip, knee, or spine; (c) hip or knee arthroplasty performed more than one year prior to enrollment; (d) osteoporosis under treatment with no fractures in the past year; (e) medically controlled primary hypertension; (f) stable type 2 diabetes; (g) no participation in a rehabilitation program in the last 12 months; (h) balanced neuropsychological status; (i) no injuries in the past 6 months; (j) ability to respond to a self-report questionnaire.

Exclusion criteria: Patients were excluded if they met any of the following: (a) dependent living situation or recent orthopedic surgery, trauma, or immobilization within the past 3 months; (b) difficulty in performing daily living activities such as bathing, dressing, or mobility; (c) diagnosed psychiatric disorders confirmed by a medical specialist; (d) major comorbidities including cancer, stroke, parkinsonism, autoimmune or hematologic disorders, heart failure, COPD, cirrhosis, or chronic kidney disease; (e) endocrine disorders affecting calcium metabolism (excluding osteoporosis); (f) chronic use of corticosteroids; (g) non-compliance or unwillingness to participate in daily rehabilitation activities (for CG).

#### 2.2.2. Recruitment and Initial Screening

Initially, 360 consecutive patients with chronic joint and musculoskeletal disorders were assessed at the Department of Physical Medicine and Rehabilitation, Filantropia Hospital, Craiova. Chronic pain was confirmed based on the patient’s response to the question: “In the past six months, have you felt pain in any area of your body that persisted daily for a duration of three months or longer?” [26]. We applied the ESPEN/EASO diagnostic algorithm for SO, which includes three steps: case-finding via the Strength, Assistance with walking, Rise from a chair, Climb stairs, and Falls questionnaire (SARC-F), confirmation through physical performance or strength testing, and body composition analysis [27].

All patients underwent anthropometric measurements and SARC-F screening (cut-off score ≥ 4) [28]. Measurements (weight, height, Body Mass Index [BMI]) were conducted using standardized equipment (Fazzini SRL, Buccinasco, Italy), by the same evaluator, and performed twice for reliability. BMI was calculated using the following formula: weight (kg)/height (m)^2^ [29].

#### 2.2.3. Sarcopenia Diagnosis

Out of the primary screened patients, 145 presented both elevated BMI (>30 kg/m^2^) and SARC-F ≥ 4. These patients proceeded to further diagnostic evaluation in two stages:

- Step 1—Muscle Strength and Physical Performance:
  - Handgrip strength (HGS) was measured with a Saehan SH5008 dynamometer. Patients were instructed to stand upright with the dynamometer beside them, and the elbow was flexed to a 90° angle. Maximal isometric effort for 5 s was performed three times for the dominant side. The best of all attempts was used for study; the cut-off value for men: <27 kg; for women: <16 kg [30];
  - Gait velocity was assessed by instructing the participants to traverse an 8 m path. The timing commenced when the participant crossed the 1 m mark and concluded at the 7 m mark, covering a span of 6 m. This procedure was repeated twice, and the superior performance was noted. A threshold speed of 0.8 m per second was established as the criterion [31].

- Step 2—Body Composition Analysis:
  - Patients with reduced HGS or gait speed underwent Bioelectrical Impedance Analysis (BIA) using the Omron BF511 device. Parameters included skeletal muscle mass (SMM), BF%, and SMMI. To minimize variability and ensure accurate results, the bioimpedance analysis was conducted under standardized conditions: at the same time of day, following similar food intake patterns, at least a few hours after any physical activity. The measurements were performed by the same evaluator, and results were interpreted using standardized values adjusted for age and sex [32];
  - Pathological values were defined as SMM < 24% of body weight or SMMI < 7 kg/m^2^ for men and <5.7 kg/m^2^ for women [33];
  - Obesity was defined as body fat > 60th percentile: >27% for men and >38% for women [34].

The combination of abnormal muscle function and composition confirmed SO. A total of 82 patients were diagnosed with stage I SO, without complications, as defined by exclusion of severe metabolic or functional impairments [35].

#### 2.2.4. Clinical and Functional Assessments

- Participants underwent laboratory analysis, including C-reactive protein, fibrinogen, lipid profile, adiponectin, leptin, and TNF-α using ELISA kits (Biovendor R&D, Brno, Czech Republic);
- Physical performance was assessed with SPPB, evaluating balance, gait speed, and chair rise ability. The SPPB is used to objectively assess lower limb function in older adults through three tests: static body balance, lower limb muscle strength (chair stand test), and gait (4 m gait walk) [36]. For balance, the patient had to maintain three different positions for 10 s: (a) Feet together, (b) semi-tandem position (the ankle of one foot behind the joint of the other foot), and (c) tandem position (the toes of one foot directly behind the heel of the other foot and touching it). For the chair stand test and 4 m gait walk, the same procedure was followed as explained above. For each of the tests, scores range from 0 to 4 points, with a maximum score on the instrument of 12 points. A higher score indicated a better physical performance. A score less than or equal to 8 is indicative of severe sarcopenia [37];
- Pain severity was evaluated with NRS, a validated 0–10 scale (0 = no pain; 10 = worst imaginable pain) [38];
- To assess central sensitization, PPT was measured bilaterally at the lumbar region using an Algometer II (SBMedic Electronics, Sweden). The probe (1 cm^2^) was applied perpendicularly with pressure increasing at ~1 N/s until pain was reported. The mean of three trials at 30 s intervals was recorded [39,40]. The lumbar location was selected due to literature evidence linking chronic low back pain with muscle degradation and systemic sensitization mechanisms in musculoskeletal disorders [41].

#### 2.2.5. Sarcopenia-Specific Quality of Life

To assess sarcopenia-related quality of life, we used the SarQoL questionnaire (SarQoL)—a validated tool designed for sarcopenic populations [42]. It contains 55 items across 7 dimensions: physical/mental health, mobility, body composition, function, daily activities, leisure, and concerns. The majority of the questions (19 out of 22) employ a Likert scale to measure frequency or intensity, allowing participants to select the option that best describes their experience. Each dimension is rated on a scale from 0 to 100, with an aggregate score being computed. Higher scores indicate a better HRQoL [42]. Scores range from 0 to 100, with higher values indicating better quality of life. Patients completed the official Romanian version of the SarQoL. Scoring was performed using the algorithm provided by the developers [43].

### 2.3. Study Treatment

The rehabilitation program was conducted under the supervision of a certified physiotherapist. A comprehensive baseline evaluation was first performed by a rehabilitation physician and included patient medical history, clinical examination, and assessment of functional status.

All patients, regardless of group assignment, received initial general recommendations structured around three core pillars:

- Patient education and support: Counseling regarding the impact of obesity and sarcopenia on functional health, and the pivotal role of physical activity in improving body composition and reducing chronic pain;
- Nutritional support: Emphasis on protein intake (1.2–1.5 g/kg body weight/day), distributed evenly across meals to enhance muscle protein synthesis. Patients were advised on tailored dietary strategies to simultaneously address sarcopenia and obesity to prevent further decompensations [44];
- Pharmacological treatment: Supplementation with vitamin D and calcium was recommended to support musculoskeletal integrity. The administration of additional analgesic treatment was not permitted during the study period.

Adherence to the full rehabilitation program—including physical therapy sessions and home training—was specific to SG, while CG received only general advice and nutritional support.

**Figure 2:**
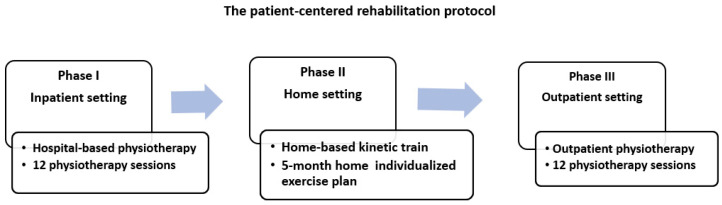
The patient-centered rehabilitation protocol.

Rehabilitation Program Structure:

The patient-centered rehabilitation protocol (Figure 2) consisted of three distinct stages:

1. Phase I (hospital-based physiotherapy): 12 physiotherapy sessions conducted in an inpatient setting;
2. Phase II (home-based kinetic training): 5-month individualized exercise plan performed at home, with monthly in-person monitoring by outpatient services;
3. Phase III (outpatient physiotherapy): A second round of 12 physiotherapy sessions provided through the outpatient clinic.

The interventions were selected based on current literature addressing sarcopenia, obesity, and chronic pain—although comprehensive studies integrating all three conditions are limited and often vary in methodology and sample size [45,46,47,48,49,50,51,52]. This evidence-informed strategy enabled us to design a tailored, effective, and scalable protocol for managing this complex clinical profile.

The selection of physiotherapeutic techniques in the inpatient phase was based on their evidence-supported physiological effects and compatibility with the clinical profiles of elderly patients with SO and chronic pain. Each modality was chosen for its specific contribution to muscular, metabolic, and analgesic recovery goals.

- Pulsed Electromagnetic Field Therapy (PEMF) and focused magnetic field (FMF) were incorporated due to their anti-inflammatory, analgesic, and regenerative effects at the cellular level. PEMF promotes microcirculation, supports soft tissue repair, and modulates inflammatory cascades, which is particularly relevant in chronic musculoskeletal conditions. FMF application allows focused energy delivery to targeted areas such as thighs or lumbar spine, enhancing localized healing responses [53,54,55];
- Electrical muscle stimulation (EMS) was employed to enhance skeletal muscle function through the stimulation of type II muscle fibers, which are typically lost during sarcopenia. Low-frequency stimulation focused on improving neuromuscular activation and strength, while higher-frequency protocols promoted muscle mass preservation. The method is especially useful for patients with limited physical capacity, offering muscle engagement without joint overload [56,57,58];
- Low-Level Laser Therapy (LLLT) was selected for its myoregenerative and anti-inflammatory properties. The use of infrared wavelengths (808 nm) has demonstrated beneficial effects on mitochondrial function and ATP synthesis, supporting muscle tissue regeneration and reducing oxidative stress—both relevant in the treatment of sarcopenia-related muscle degeneration [59,60,61,62];
- Deep oscillation therapy via manual applicator was chosen for its dual-phase therapeutic impact: an initial high-frequency phase (100 Hz) for analgesia and muscle relaxation, followed by a low-frequency range (5–25 Hz) to stimulate local metabolism and enhance lymphatic flow, contributing to pain relief and functional recovery in soft tissues [62,63,64];
- Kinesiotherapy, delivered in the form of resistance training, balance and gait exercises, and aerobic training, was integrated to comprehensively address muscle endurance, postural control, cardiovascular health, and mobility [65,66,67,68]. The protocols emphasized low-impact, progressive load strategies to prevent pain exacerbation and ensure safety, particularly considering comorbid low back pain in the target population.

Details of the physical therapy modalities and kinetic exercise routines applied in Phase I and Phase III are provided in Table 1.

**Table 1:** Description of the physiotherapy techniques and kinesiotherapy applied during Phases I and III.

	Components	Description
The first stage (2 weeks) 12 sessions of physio- and kinesiotherapy Inpatient	Pulsed Electromagnetic Field Therapy (PEMF) with focused magnetic field (FMF) (BTL-5000 Czech Republic by BTL Industries (Prague, Czech Republic))	Lumbar solenoid (60 cm) and disc applicator (13 × 13 × 3 cm) on both thighs (BTL-239-1). Key parameters: Rectangular pulses with 10 Hz frequency, 50 µT intensity. For FMF, applied through disc applicator, the total intensity used was 128 mT. 30 min per session, twice daily (morning session, after waking up, at 9 a.m. and evening session before going to bed at 7 p.m.).
	Electrical muscle stimulation (EMS) of the lumbar region, quadriceps muscles, and calf muscles (Endomed 482, device series 42.400, Enraf-Nonius, Netherlands)	Biphasic rectangular pulses, pulse frequency 100 Hz (Hertz), in steps of 1 Hz, pulse-width of 150 µs, 2 milliseconds phase duration, 30 Hz current frequency were used. Established load ratio of 3 s of current followed by 3 s of rest (ratio 1:1). Intensity: 10 mA (adjusted to patient comfort, typically to the point of visible muscle contraction without causing pain). 30 min daily session of EMS for 6 days each week using two pairs of 10 cm × 15 cm carbon rubber electrodes (150 cm^2^), one for each side, stimulated both muscle groups and lumbar region, 10 min each region.
	Low-Level Laser Therapy (LLLT) 20 min, daily (ASTAR PhysioGo 500I/501I Poland, PhysioGo series)	Wavelength of 808 nm, a power of 100 mW, an energy dosage of 7 J/cm^2^, and energy per point of 0.003 J. Applied to shoulder girdle muscles, quadriceps, and major gluteus, left and right.
	Deep oscillation therapy with manual applicator Personal device DOP1.1.—INDIVID—Physiomed (device series—2442007) Germany, Physiomed Elektromedizin AG	First, 10 min—high frequency 100 Hz—in the lumbosacral region. Then, 10 min—low frequency 5–25 Hz. 5 cm oscillator head applied in both thigh muscles. Total: 30 min daily.
	Kinesiotherapy: - Resistance exercises, daily, 15 min - Balance and gait training, daily, 15 min - Low-intensity aerobic training (endurance training) performed at 40–60% of maximum heart rate, according to the equation ((220 − age) × 0.65), three times a week, 40 min	***Resistance exercises (strength training)—performed a.m.*** Muscle groups: Focus on low-impact exercises that target both the upper and lower body but are gentle on the back. Include exercises that strengthen the core, as this can help alleviate low back pain. Load: Start with very light weights or body-weight exercises, particularly for the lower back and core. The initial load should be about 20–30% of baseline strength to avoid straining the back. Repetitions and sets: Perform higher repetitions (10–15) at a lower intensity to focus on muscle endurance, crucial for sarcopenia. Keep to 2–3 sets to avoid fatigue. Rest intervals: Extend rest intervals to 120–180 s between sets to ensure full recovery, especially important for patients with low back pain. Equipment: Utilize resistance bands (TheraBand Latex Resistance Bands) and body-weight exercises to minimize stress on the lumbar spine. ***Balance and gait training***—***performed in a.m.*** Supportive equipment: Incorporate balance exercises that can be performed while seated or holding onto a stable object to reduce the risk of falls and lower back strain. Duration and intensity: Start with short sessions of simple balance exercises (mono- and bipedal walking), gradually increasing the complexity as the patient’s balance improves. ***Endurance training (aerobic training)—performed in p.m.*** Many repetitions, low resistance, using large muscle groups. Exercise selection: Choose non-weight-bearing activities such as walking or cycling on a recumbent bike to reduce impact on the back and joints. Intensity and duration: Begin with very low intensity (20–30% of maximum heart rate) for short durations (5 min). Gradually increase as tolerated without exacerbating pain. Monitoring: Keep a close watch on the patient’s back pain during exercises, adjusting the program as needed to avoid discomfort. Warm-up and cool-down: Emphasize gentle stretching, particularly of the lower back and core muscles, and include breathing exercises to help relax the muscles and reduce pain.
The second stage (5 months)—see Table 2 Home training kinetic program with monthly monitoring through the outpatient service		
The third stage (2 weeks) 12 physio- and kinesiotherapy sessions Outpatient	Pulsed Electromagnetic Field Therapy (PEMF) with focused magnetic field (FMF) (BTL-5000 Czech Republic by BTL Industries)	Lumbar solenoid (60 cm) and disc applicator (13 × 13 × 3 cm) on both thighs (BTL-239-1). Key parameters: Rectangular pulses with 10 Hz frequency, 50 µT intensity. For FMF, applied through disc applicator, the total intensity used was 128 mT. 30 min per session, twice daily (morning session, after waking up, at 9 a.m. and evening session before going to bed at 7 p.m.)
	Low-Level Laser Therapy (LLLT) 10 min, daily ASTAR PhysioGo 500I/501I Poland, PhysioGo series	Wavelength of 660 nm, a power of 100 mW, an energy dosage of 5 J/cm^2^, and energy per point of 0.003 J.
	Deep oscillation—therapy with manual applicator Personal device DOP1.1.—INDIVID—Physiomed (device series—2442007) Germany, Physiomed Elektromedizin AG	First, 10 min—high frequency at 100 Hz. Then, 10 min—low frequency at 5–25 Hz. 5 cm oscillator head applied in both thigh muscles. Total: 30 min daily.
	Kinesiotherapy - Functional and balance training - Aerobic endurance training (30–40 min)	Implement balance training such as standing on one foot, walking heel-to-toe, or using balance boards to reduce fall risk and improve body coordination. (10 min) Practice functional movements that mimic daily activities, such as stepping up and down a stair, sitting down and standing up from a chair, and carrying weights to simulate grocery bags (10 min). Pedaling on the elliptical bike. Begin with short durations (10–15 min) and gradually increase to 20 min.

Home-based training was designed to span 5 months. Patients were contacted regularly via telephone by the physiotherapist who had initiated their program. This communication allowed for continuous monitoring, encouragement, and adherence validation.

During these follow-ups, the following principles were reinforced:

- Progression: Gradual increase in exercise intensity and duration, adjusted to each patient’s tolerance;
- Safety: Emphasis on using support or supervision during training to prevent falls or injuries;
- Hydration and nutrition: Reminders to maintain adequate hydration and consume a balanced, protein-rich diet to support recovery and muscle mass preservation.

**Table 2:** Description of the kinetic exercise routines performed during home-based training (Phase II).

Day	Type of Training	Activities	Duration
Day 1	Aerobic training	Warm-up (10 min): Gentle stretching and slow walking or stationary cycling Main activity (20 min): Walking on flat surface or treadmill Cool-down (10 min): Slow walking and stretching	40 min
Day 2	Resistance training	Warm-up (10 min): Light stretching and mobility exercises Focus on gentle movements; avoid any that cause discomfort Main activity (20 min): - Seated leg press—adjust weight for comfort, ensure back support; - Arm curls with resistance bands—use low-resistance bands; perform seated if necessary; - Chest press—use light resistance, perform seated with back support. Cool-down (10 min): Gentle stretching for chest, arms, legs; hold stretches for 6–10 s for each part.	40 min
Day 3	Balance training	Warm-up (10 min): Gentle stretching of the upper and lower body. To prepare the muscles and joints for exercise, reducing the risk of injury. Ensure that stretches are performed gently and within a comfortable range of motion, especially for the lower back. Include dynamic stretches that mimic the movements of the main activity to better prepare the body. Main activity (20 min): *Seated leg extensions* to strengthen the quadriceps, which are crucial for knee stability and balance. Sit in a sturdy chair, extend one leg out straight, hold for a few seconds, and then lower it back down. Repeat with the other leg for 6 repetitions, 30–60 s rest periods, perform slowly, ensuring each leg is extended fully before lowering *Chair squats* to strengthen the lower body, including the hips, thighs, and buttocks, which support balance. Stand in front of a chair with feet hip-width apart. Slowly bend the knees and lower the body as if to sit, touch the chair lightly, then stand back up. 6 repetitions, 30–60 s rest periods, stand and lower to just touch the chair, then stand back up slowly *Wall push-ups* to enhance upper body strength, which helps in maintaining overall stability. Stand facing a wall, place hands on the wall at shoulder width and level, then bend the elbows to bring the chest towards the wall and push back to the starting position. 6 repetitions, 30–60 s rest periods, adjust the distance from the wall to maintain comfort; push up slowly. Cool-down (10 min): Gentle stretching focusing on the legs and lower back. To relax the muscles and gradually return the heart rate to normal, preventing muscle stiffness. Include stretches that specifically target the lower back, such as knee-to-chest stretches or pelvic tilts, to alleviate any tension built up during the exercise. Ensure that all movements are slow and controlled.	40 min
Day 4	Rest day	Activity: Light walking or leisure activities (gardening, shopping, light housework).	40–60 min
Day 5	Combined aerobic and light resistance training	Warm-up (10 min): See Day 3. Main Activity (40 min): Cycling on a stationary bike, light resistance circuit. Cool-down (10 min): See Day 3.	60 min
Day 6	Flexibility training	Warm-up (10 min): Light cardiovascular exercise like walking or stationary cycling at a very low intensity. Main activity (20 min): Dynamic stretches: Leg swings and arm circles to improve range of motion. Static stretches: Hold stretches for each major muscle group for 20–30 s, such as hamstring and quadriceps stretches and arm stretches. Cool-down (10 min): Deep breathing and relaxation techniques to enhance muscle relaxation.	40 min
Day 7	Rest day	Activity: Light, non-strenuous activities such as walking around the home or gardening, gentle walking in a park with low-intensity movements	30–60 min

### 2.4. Statistical Analysis

Statistical analyses were conducted using Microsoft Excel 365 (Microsoft Corporation, Redmond, WA, USA); IBM SPSS Statistics for Windows, Version 28.0 (IBM Corp., Armonk, NY, USA); and XLSTAT add-on for Microsoft Excel (Addinsoft SARL, Paris, France). All data were initially recorded and organized in Excel spreadsheets before being processed in SPSS and XLSTAT to evaluate intra- and inter-group differences and correlations.

Quantitative variables were expressed as mean ± standard deviation (SD) and median (25th–75th percentiles), while qualitative variables were reported as absolute numbers and percentages.

Normality assessment: The Shapiro–Wilk test was applied to assess the distribution of continuous variables (SarQoL, SPPB, NRS, and PPT). As most variables did not follow a normal distribution (*p* < 0.05), non-parametric statistical methods were selected for the analyses.

Within-group comparisons: To evaluate changes between T1 and T2 timepoints for each group, the Wilcoxon signed-rank test was employed. This test assessed whether the differences in clinical scores before and after the intervention were statistically significant.

Between-group comparisons: The Mann–Whitney U test was used to compare SG and CG at each time point. This non-parametric test was chosen due to the non-normal distribution of data.

Correlation analysis: The Spearman rank correlation coefficient was applied to explore associations between outcome variables and demographic or clinical parameters.

Effect size and power analysis: Effect sizes were calculated for each outcome variable using the following formula, adjusted for non-parametric tests:

N=Z α2  +Zβd2  ×1E

In this formula, N is the required sample size per group; Zα/2 = 1.96 (alpha = 0.05) is the z-score corresponding to the desired significance level (alpha); Zβ = 0.84 (for 80% power) is the z-score corresponding to the desired power (1—beta); d = 0.843 is the estimated effect size (the calculated average effect size of approximately 0.843 indicates a strong relationship between the specified parameters- SarQoL, SPPB, SMMI—in the SG); and E is the efficiency of the non-parametric test relative to the parametric test. For the Wilcoxon signed-rank test, this is often estimated at around 0.864.

The calculated effect sizes for SG are as follows: NRS: −3.5767; PPT: 0.8891; SPPB: 1.9356; and SarQoL: 1.2529. The CG effect sizes are NRS: −0.7382; PPT: 0.0; SPPB: 0.3828; and SarQoL: −0.0266.

These effect sizes indicate the magnitude of change between two time points for each parameter within each group. A higher absolute value of the effect size indicates a more substantial change. For instance, the SPPB shows a significant change in the SG compared to the CG, suggesting a stronger intervention effect or more substantial improvement in physical performance in the SG.

The negative effect size for NRS in the SG suggests a reduction in pain ratings, which is typically a positive outcome in clinical settings. The zero effect size for PPT in the CG indicates no change between the two time points measured.

These calculations provide valuable insights into the effectiveness of interventions or conditions being studied in each group, particularly in terms of pain management, physical performance, and quality of life.

Given the effect sizes calculated for the parameters like NRS, PPT, SPPB, and SarQoL, the required sample size for the Wilcoxon signed-rank test is approximately 12 participants per group. This conservative estimate ensures that the earlier calculations align with the significant effect sizes observed and accounts for desired power and test efficiency.

### 2.5. Ethics Approval

This study was conducted with careful consideration for the safety, autonomy, and overall well-being of all participants, recognizing the increased vulnerability of individuals affected by SO and chronic musculoskeletal conditions. Prior to enrollment, each participant received detailed, comprehensible, and age-appropriate information regarding this study’s purpose, potential risks and benefits, and the procedures implemented to ensure strict confidentiality of personal data in accordance with applicable data protection legislation.

Participants were explicitly informed of their right to withdraw from this study at any time without any obligation to justify their decision and without incurring any prejudice to their ongoing care.

All participants provided written informed consent after a thorough explanation of the study protocol, and any additional questions were addressed by the research team to ensure full understanding and voluntary agreement.

The study protocol complied with the ethical principles set forth in the Declaration of Helsinki and adhered to Good Clinical Practice (GCP) guidelines. Ethical approval was obtained from the Ethics Committee of the University of Medicine and Pharmacy of Craiova, under approval number 204/20 September 2023.

## 3. Results

### 3.1. Baseline Patient Characteristics

The baseline demographic, anthropometric, and clinical characteristics of both study and control groups are summarized in Table 3.

Overall, the groups were largely comparable, with no statistically significant differences in most numeric or categorical variables, except for weight and SarQoL, where significant differences were identified.

Demographically, participants in both groups had a similar age distribution, with the study group averaging 72.66 years and the control group 72.92 years (difference: 0.26 years). Gender distribution and place of residence also showed no statistically significant differences (*p* > 0.05, chi-square), confirming demographic homogeneity.

Anthropometric analysis revealed that the study group had a lower average weight (81.11 kg) compared to the control group (84.14 kg; *p* < 0.05). However, the average height was nearly identical between the two groups, resulting in a comparable BMI, with no statistically significant differences (*p* > 0.05). Body fat percentage was slightly lower in the study group (45.09%) compared to the control group (46.71%), but this difference was not significant.

Functionally, both groups showed nearly identical scores in the SPPB and SMMI, indicating similar levels of physical performance and sarcopenic status at baseline. Pain assessment using NRS showed lower values in the study group (6.94) compared to the control group (8.72), while PPT scores were also lower in the study group (69.31 vs. 72.36), suggesting less perceived pain; however, these differences were not statistically significant (*p* > 0.05).

Regarding quality of life, measured by SarQoL, the study group reported a significantly lower score compared to the control group (mean difference = 2.77; *p* < 0.05). This might reflect a higher motivation among study group participants to engage actively in the rehabilitation program.

These baseline findings confirm that, except for weight and SarQoL, the two groups were well matched. This supports the validity of comparing outcomes post-intervention, minimizing the risk that differences in results are due to pre-existing disparities.

### 3.2. Study Group: Time Evolution

Table 4 presents the evolution of parameters between T1 and T2, with a focus on SarQoL, SPPB, NRS, and PPT. All variables showed statistically significant changes post-intervention, confirmed using the Wilcoxon signed-rank test.

The test results showed the following:

- SarQoL: Test statistic = 55.0, *p* = 3.25 × 10^−6^;
- SPPB: Test statistic = 0.0, *p* = 4.99 × 10^−7^;
- NRS: Test statistic = 0.0, *p* = 5.82 × 10^−11^;
- PPT: Test statistic = 11.0, *p* = 3.20 × 10^−9^.

**Figure 3:**
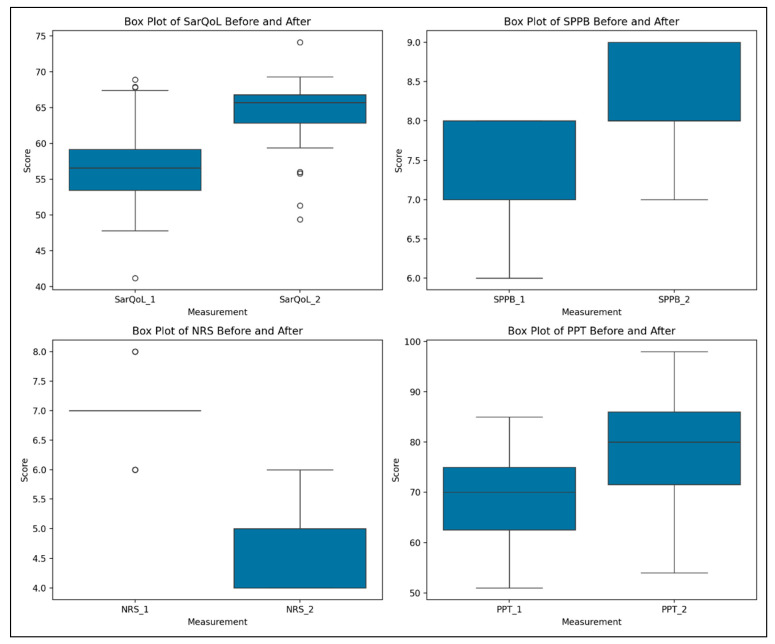
The box plots for the variables SarQoL, SPPB, NRS, and PPT, showing their distributions before and after the intervention in SG. 1 = T1; 2 = T2; SarQoL = SarQoL questionnaire; SPPB = Short Physical Performance Battery; NRS = Numeric Rating Scale for pain; PPT = Pressure Pain Threshold.

**Figure 4:**
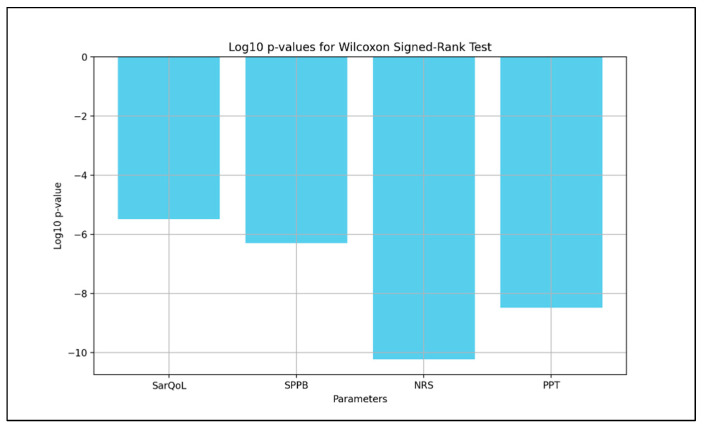
Logarithmic transformation of *p*-values from the Wilcoxon signed-rank test conducted on four key parameters: SarQoL, SPPB, NRS, and PPT for SG. SarQoL = SarQoL questionnaire; SPPB = Short Physical Performance Battery; NRS = Numeric Rating Scale for pain; PPT = Pressure Pain Threshold.

These results indicate strong evidence against the null hypothesis and support the positive effects of the rehabilitation program. A log10 transformation of *p*-values was applied to enhance interpretability, and the results are illustrated below (Figure 3 and Figure 4).

When stratifying by sex and environment, distinct trends emerged (Table 5).

- SarQoL: Highest improvement in urban men (+9.99), lowest in rural men (+4.94);
- SPPB: Relatively stable gains across all subgroups;
- NRS: Moderate reduction in all subgroups;
- PPT: Highest gains in rural men (+15.60).

These subgroup differences suggest that environmental setting and gender influence treatment response, particularly in SarQoL and PPT.

**Figure 5:**
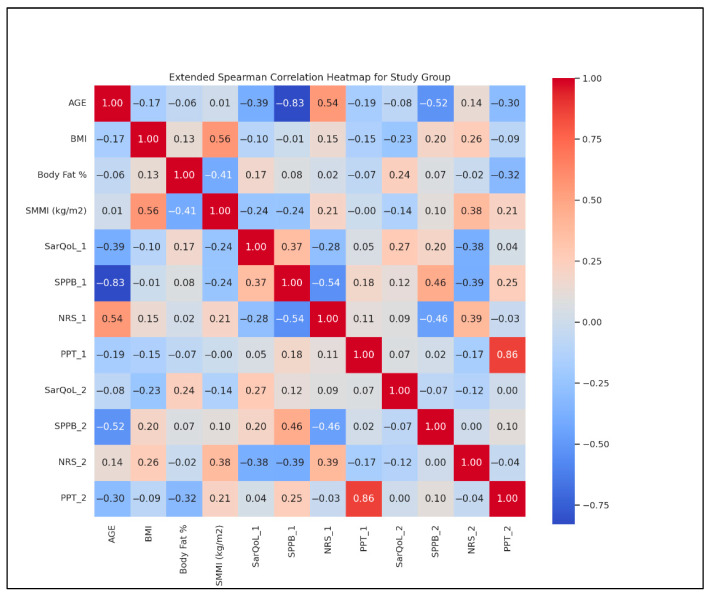
Spearman correlation heatmap for the SG. 1 = T1; 2 = T2; SarQoL = SarQoL questionnaire; SPPB = Short Physical Performance Battery; NRS = Numeric Rating Scale for pain; PPT = Pressure Pain Threshold.

To further assess interrelationships, Spearman correlations were calculated pre-intervention (Figure 5).

Key findings:

- Age and BMI: r = 0.45;
- BMI and BF %: r = 0.75;
- SMMI and SPPB: r = −0.30;
- SarQoL and NRS: r = −0.55;
- PPT and NRS: r = −0.65.
- Heatmap analysis also showed the following:
- Strengthened SarQoL-SPPB correlation from T1 (r = 0.45) to T2 (r = 0.65);
- Weakened BMI-NRS correlation from T1 (r = −0.30) to T2 (r = −0.50).

These findings reinforce the interconnected nature of physical function, pain, and quality of life in sarcopenia and highlight the efficacy of rehabilitation tailored by demographic profiles.

### 3.3. Control Group: Time Evolution

In CG, the same parameters were analyzed over time (T1–T2) to assess the natural progression without an active rehabilitation program. The results, detailed in Table 6, indicate that there were no statistically significant improvements in any of the measured outcomes.

Specifically, we found the following:

- SarQoL scores showed minor, non-significant changes (*p* > 0.05);
- SPPB values remained stable between the two time points (*p* > 0.05);
- NRS for pain and PPT also demonstrated no statistically significant variations (*p* > 0.05).

**Figure 6:**
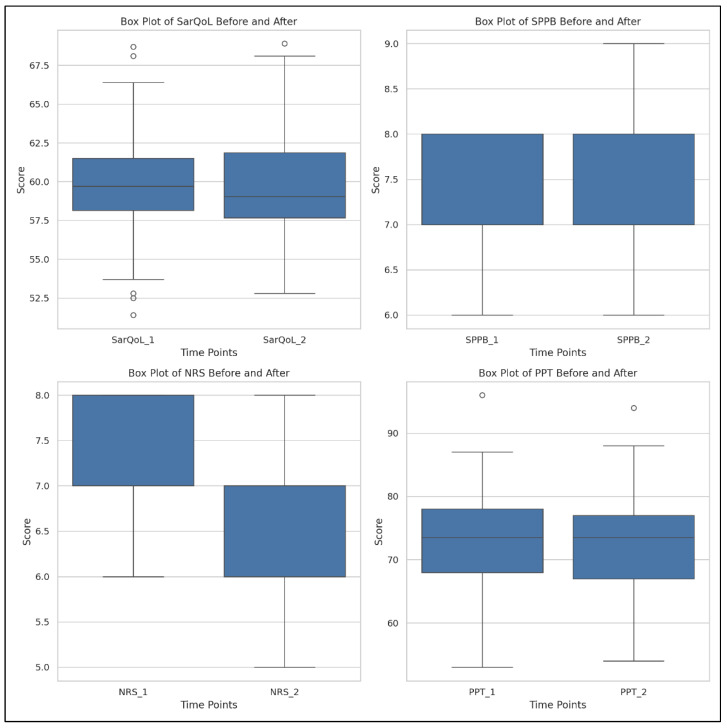
The box plots for the variables SarQoL, SPPB, NRS, and PPT, showing their distributions before and after the intervention in the control group. 1 = T1; 2 = T2; SarQoL = SarQoL questionnaire; SPPB = Short Physical Performance Battery; NRS = Numeric Rating Scale for pain; PPT = Pressure Pain Threshold.

A visual overview of these unchanged parameters is provided in Figure 6, which presents box plots for SarQoL, SPPB, NRS, and PPT measured in the CG at T1 and T2. The distributions indicate minimal variation across time points, supporting the statistical results that revealed no significant evolution in any of the studied variables.

To further explore subgroup dynamics in the control group, we analyzed outcome variations stratified by sex and environment. These results are summarized in Table 7.

Across all subgroups, the observed changes from T1 to T2 were minor and mostly statistically non-significant. For instance, rural women showed a statistically significant reduction in NRS scores (*p* = 0.01), but other changes (in SarQoL, SPPB, and PPT) were not significant across subgroups (*p* > 0.05). These results suggest that in the absence of a structured rehabilitation program, the natural evolution of symptoms remains relatively stable across demographic contexts.

**Figure 7:**
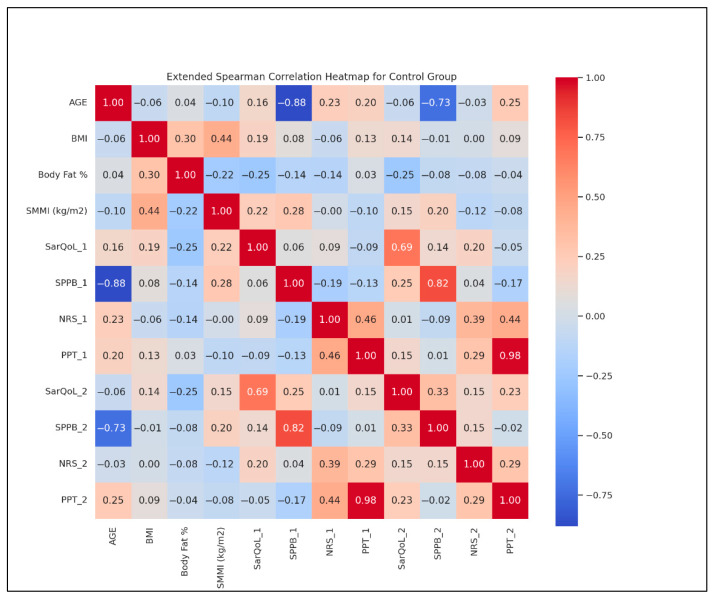
Spearman correlation heatmap for the control group. 1 = T1; 2 = T2; SarQoL = SarQoL questionnaire; SPPB = Short Physical Performance Battery; NRS = Numeric Rating Scale for pain; PPT = Pressure Pain Threshold.

To complement the analysis, Figure 7 displays the extended Spearman correlation heatmap for the control group. This visualization illustrates the strength and direction of associations between all measured variables at T1 and T2.

Notable findings include strong positive correlations between BMI and BF % (r = 0.75), as well as a marked inverse correlation between age and SPPB at T1 (r = −0.88) and T2 (r = −0.73). The consistent correlation between PPT and NRS (r = −0.65) supports the clinical observation that pain threshold is inversely related to pain perception.

These results collectively reinforce the stability of CG across multiple outcome domains and emphasize the distinct benefits observed in SG.

### 3.4. Study Group Versus Control Group

**Figure 8:**
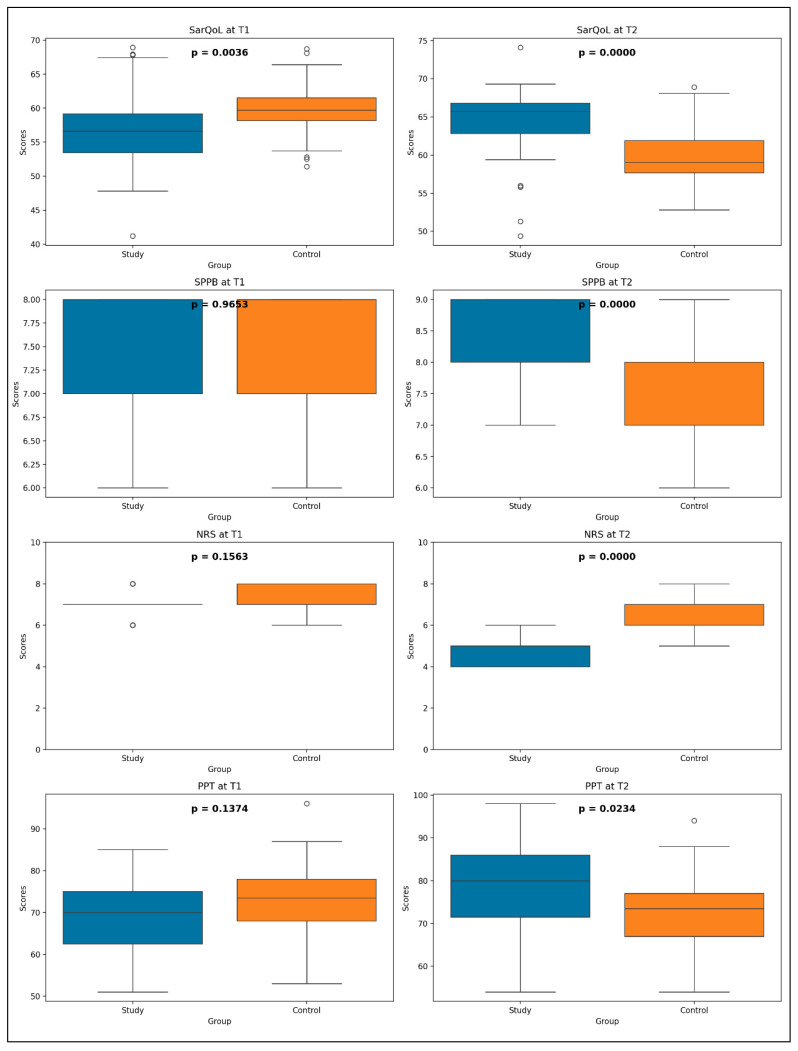
Comparative analysis of SG and CG across key health parameters (SarQol, SPPB, NRS, PPT) at two time points: T1 and T2. SarQoL = SarQoL questionnaire; SPPB = Short Physical Performance Battery; NRS = Numeric Rating Scale for pain; PPT = Pressure Pain Threshold.

To evaluate the effectiveness of the rehabilitation intervention, a comparative analysis was conducted between SG and CG at T1 and T2 (Figure 8). The Mann–Whitney U test was used to compare SarQoL, SPPB, NRS, and PPT between the two groups. The test statistics and corresponding *p*-values offer insight into the evolution of these health outcomes.

At T1, the only statistically significant difference observed was for SarQoL (U = 376.5, *p* = 0.0036), suggesting an initial disparity in quality of life related to sarcopenia. No significant differences were found for the following:

- SPPB (U = 634.0, *p* = 0.9653);
- NRS (U = 517.0, *p* = 0.1563);
- PPT (U = 500.5, *p* = 0.1374).

These findings indicate that, prior to the intervention, both groups were functionally similar, aside from perceived quality of life.

At T2, statistically significant differences were recorded across all four variables:

- SarQoL: U = 984.5, *p* < 0.0001;
- SPPB: U = 1072.0, *p* < 0.0001;
- NRS: U = 31.0, *p* < 0.0001;
- PPT: U = 827.5, *p* = 0.0234.

These results strongly suggest that the rehabilitation program produced meaningful improvements in sarcopenia-related quality of life, physical function, pain perception, and pressure pain threshold, compared to the CG.

The significant differences in SarQoL scores at both time points underscore the potential impact of the studied interventions on quality of life in sarcopenic individuals. The absence of significant differences at T1 for SPPB, NRS, and PPT—followed by their emergence at T2—demonstrates a delayed but robust treatment effect.

These findings emphasize the importance of longitudinal monitoring in capturing the full benefits of rehabilitation strategies, particularly when evaluating complex conditions like sarcopenia that may involve gradual recovery in function and symptom relief.

## 4. Discussion

This prospective controlled trial without randomization is the first to evaluate a comprehensive rehabilitation program specifically designed for patients with the co-occurrence of SO and chronic pain—a triad increasingly recognized in clinical research. The previous literature addressed only dyadic relationships such as sarcopenia–obesity [69,70,71,72], sarcopenia–pain [73,74], or obesity–pain [75]. Our findings confirm the feasibility and clinical effectiveness of a multimodal kinetic and physiotherapeutic intervention, leading to significant improvements in pain management, physical performance, and quality of life. This study integrated multiple clinical endpoints reflecting the multidimensional impact of sarcopenic obesity with chronic pain. While the scope may appear broad, the selected outcomes are interdependent and reflect the reality of clinical practice. While the protocol employed a multimodal approach that integrates electrotherapy and kinetic therapy elements, it was intentionally designed as a whole-system intervention model, reflecting clinical realities where synergistic effects are common. The current study did not aim to dissect the individual contributions of each component, but future investigations may employ factorial designs for that purpose.

The reduced pain thresholds identified in our patients with sarcopenic obesity and chronic pain support the presence of central sensitization mechanisms in this population. These findings are in line with our previous research on post-COVID-19 fibromyalgia patients [76], where similar alterations in pain processing were documented. In SG, pain intensity decreased significantly both statistically and clinically, with a very large effect size, while the substantial increase in PPT further confirms the effectiveness of the applied rehabilitation program in modulating central sensitization. These results support previously described associations between chronic pain and sarcopenia [77,78,79] and reinforce the importance of multimodal pain treatment strategies [80]. The study results are consistent with findings by Veronese et al. [79], who emphasized the bidirectional relationship between chronic pain and muscle loss. The objective use of PPT, measured via manual algometry, reflects current best practices in evaluating pain sensitization in elderly populations [81,82]. Notably, women in this study reported lower PPT values and higher pain ratings, consistent with the literature indicating sex-related differences in pain perception and coping strategies [83]. This underlines the importance of sex-specific tailoring of rehabilitation protocols.

Functional capacity, assessed through SPPB, improved significantly in SG, corresponding to a large effect size. This exceeds the minimum clinically important difference (MCID) of 1 point [84,85], underscoring the effectiveness of the intervention. Our findings are aligned with those of Ghiotto et al. [86], who reported benefits of exercise interventions in adults with sarcopenic obesity. While limited data exist on the full triad, our study extends the existing evidence base by demonstrating SPPB responsiveness in a population also suffering from chronic pain. Both groups began below the eight-point cut-off used in sarcopenia screening per EWGSOP2 guidelines [87,88], highlighting the clinical relevance of the improvements.

Quality of life improved markedly in SG, with SarQoL scores increasing from baseline to post-intervention, nearly reaching the smallest detectable difference [89,90]. The large effect size confirms the robustness of this outcome. Although SarQoL is not validated specifically for sarcopenic obesity, it remains the most appropriate disease-specific tool [42,91,92,93]. The comprehensiveness of the scale likely captured the benefits of our tailored intervention, especially in domains relating to physical health, locomotion, activities of daily living, and psychological well-being. Our findings contrast with those of den Uijl et al. [94], who reported short-term improvements without long-term maintenance in obese patients. The difference may be attributed to our program’s longer duration (6 months), its tailored structure, and its integration of physiotherapy modalities specifically adapted to the triad’s complex pathology.

Recent studies show increasing recognition of the effectiveness of physiotherapy strategies—such as PEMF, EMS, LLLT, and deep oscillation—in elderly patients with chronic conditions [95,96,97,98]. However, no study to date has proposed or evaluated their synergistic integration into a unified program for this specific patient group.

Additionally, the inclusion of a home-based kinetic training phase increased patient adherence, engagement, and real-world applicability, addressing a frequent challenge noted in other chronic pain and obesity rehabilitation studies [99].

**Practical applicability and innovation**: The applied rehabilitation protocol is notable for its multidimensional scope, longitudinal structure, and practical feasibility in clinical settings. The program addressed all three pathophysiological components of the triad: chronic pain, sarcopenia, and obesity. While the inclusion of an initial inpatient phase may present feasibility challenges in certain healthcare systems, its integration in our protocol reflects the current structure and reimbursement policies of the local rehabilitation framework. This phase was designed to support early symptom management, reinforce patient adherence, and provide structured education necessary for the effective continuation of the home-based program. From a translational perspective, this approach offers the following:

- A replicable model of multimodal rehabilitation suitable for clinical use in geriatric or outpatient rehabilitation settings;
- Educational utility in training professionals on how to integrate physiotherapy techniques with kinetic exercise regimens;
- While the present study does not isolate the effects of electrotherapy from exercise, the combination mirrors standard clinical care pathways. Further trials with active control groups receiving only one modality may clarify the individual contributions of each component;
- A framework for developing interdisciplinary chronic care pathways combining medical, nutritional, and physical interventions;
- This study has several limitations that should be acknowledged when interpreting the results. First, the sample size, although adequate for detecting changes within groups, limits the statistical power for subgroup analyses or for exploring specific profiles of treatment responders. Additionally, the absence of a factorial design prevents the isolation of the individual contributions of each therapeutic component (kinesiotherapy, electrotherapy, neurotrophic supplementation), making it difficult to determine which modality contributed most to the observed outcomes. Also, due to the hands-on nature of physical therapy interventions, a placebo control or full blinding was not feasible. Nonetheless, both groups received equal attention at baseline, including counseling and educational materials, to reduce attention bias.

Another important limitation is the lack of intermediate assessments throughout the six-month rehabilitation period. Only two evaluation timepoints were included in the study—at baseline (T1) and at the end of the protocol (T2)—which restricts the ability to identify when specific clinical or functional improvements occurred. The inclusion of additional assessments at key points during the inpatient, home-based, and outpatient phases could have provided valuable information about the timing, progression, and sustainability of treatment effects. Future studies should consider implementing a more granular evaluation timeline to better capture the dynamics of patient recovery and guide the refinement of each rehabilitation phase.

Furthermore, while adherence to the protocol was monitored through attendance records, we did not collect detailed data on participant compliance with home-based exercises or reasons for non-adherence in the 20% of cases that did not complete the protocol. The absence of adherence metrics may introduce bias and limit our understanding of the intervention’s reproducibility in routine practice.

Finally, the lack of evaluator blinding could have influenced patient performance during functional testing, despite our efforts to standardize evaluation conditions. The current trial design was not factorial and thus cannot disentangle the specific contributions of each modality. This was a conscious decision to prioritize ecological validity and implementation feasibility. Nevertheless, future randomized factorial trials are encouraged. While this limitation is common in rehabilitation trials, its potential impact on outcome measurement must be acknowledged.

**Internal validity**: This study’s internal validity was strengthened by its prospective controlled design, standardized baseline evaluations, and consistently applied interventions. The use of validated outcome tools such as the NRS, PPT, SPPB, and SarQoL contributed to minimizing measurement bias. In addition, both groups underwent identical evaluation protocols at the same time points, reducing information bias. Random allocation was not used, which may introduce selection bias, but the baseline comparability of the groups (age, gender, comorbidities) was statistically confirmed. Evaluator blinding was not implemented, which could have introduced detection bias; however, the objectivity of the tools used partially mitigates this concern. Adherence to the intervention was high, reducing attrition bias. Data collection procedures were rigorously monitored, and all participants were retained throughout the six-month program, avoiding differential dropout effects.

**External validity**: While this study was conducted in a single-center clinical setting, its external validity is supported by the broad inclusion criteria—patients with sarcopenic obesity and chronic musculoskeletal pain, a common profile in geriatric rehabilitation. The multimodal intervention combined physiotherapy and kinetic protocols adaptable to multiple clinical environments, including outpatient and home-based programs. Thus, the findings are transferable to real-world rehabilitation services, especially in similar healthcare systems with integrated care pathways. However, generalizability is limited by the sample size, lack of ethnic diversity, and exclusion of patients with severe obesity or advanced neurological disorders. Replication in larger, multicenter, and more heterogeneous populations is needed to strengthen external validity.

## 5. Conclusions

The integration of physical therapy with a kinetic program appears to be crucial in managing the dual challenges of sarcopenic obesity and chronic pain. Our study provides valuable insights into the design and implementation of effective rehabilitation programs for patients with sarcopenic obesity and chronic pain. The encouraging results underscore the importance of a personalized, multidisciplinary approach in managing these complex conditions.

Future research should expand these findings to multicenter trials with longer follow-up and explore biological, psychosocial, and behavioral mediators of the intervention effect. This would further consolidate the program’s utility in chronic multimorbidity rehabilitation.

## Figures and Tables

**Table 3:** Baseline characteristic assessment.

	Study Group (SG) 35 Patients	Control Group (CG) 36 Patients	*p*-Value	
**Age (years)**	72.65 ± 3.94	72.91 ± 3.84	*t*-test for independent samples	0.7798
**Weight (kg)**	81.11 ± 6.42	84.13 ± 4.65		**0.0259**
**Height (m)**	1.62 ± 0.07	1.63 ± 0.06		0.5488
**BMI (Kg/m^2^)**	30.78 ± 2.34	31.53 ± 1.40		0.1044
**BF %**	45.08 ± 4.62	46.71 ± 3.95		0.1162
**SMMI (kg/m^2^)**	7.07 ± 0.91	7.00 ± 0.38		0.6908
**SPPB**	7.11 ± 0.75	7.11 ± 0.70		0.9855
**SarQoL**	57.02 ± 5.94	59.79 ± 4.02		**0.0242**
**PPT (N/m^2^)**	69.31 ± 8.55	72.36 ± 9.5		0.1582
**NRS**	6.94 ± 0.63	8.72 ± 0.95		0.2730
**Urban (n, %)**	18 (52%)	18 (50%)	Chi-square statistic: 2.3034 *p*-value: 0.1291	
**Rural (n, %)**	17 (48%)	18 (50%)		
**Women (n, %)**	23 (66%)	25 (70%)	Chi-square statistic: 1.7582 *p*-value: 0.1849	
**Men (n, %)**	12 (34%)	11 (30%)		

Variables are reported as mean ± (SD) (standard deviation). n = number of subjects; % = percent of patients; BMI = Body Mass Index; body fat %; SMMI = skeletal muscle mass index; SPPB = Short Physical Performance Battery; PPT = Pressure Pain Threshold; NRS = Numeric Rating Scale for pain; SarQoL = SarQoL questionnaire.

**Table 4:** Study group parameters at initial and final assessments.

Parameters		Mean Value	SD	Min Value	25th Percentile	Median Value	75th Percentile	Max Value
**Total SarQoL**	**T1**	57.02	5.94	41.2	53.45	56.6	59.15	68.9
	**T2**	63.98	5.12	49.4	62.85	65.7	66.8	74.1
**SPPB**	**T1**	7.14	0.73	6	7	7	8	8
	**T2**	8.4	0.55	7	8	8	9	9
**NRS**	**T1**	6.94	0.63	6	7	7	7	8
	**T2**	4.65	0.63	4	4	5	5	6
**PPT**	**T1**	69.31	8.55	51	62.5	70	75	85
	**T2**	78.05	10.96	54	71.5	80	86	98

T1 = initial; T2 = final; SarQoL = SarQoL questionnaire; SPPB = Short Physical Performance Battery; NRS = Numeric Rating Scale for pain; PPT = Pressure Pain Threshold.

**Table 5:** Mean differences between initial and final measurements for SarQoL, SPPB, NRS, and PPT, respectively, by sex and environment in SG (*p*-values from the Wilcoxon signed-rank tests).

Sex	Environment	SarQoL_diff	SPPB_diff	NRS_diff	PPT_diff
**Women**	Urban	6.24 (*p* = 0.03)	1.27 (*p* = 0.006)	−2.36 (*p* < 0.001)	7.36 (*p* < 0.001)
**Women**	Rural	6.67 (*p* = 0.02)	1.33 (*p* = 0.002)	−2.25 (*p* < 0.001)	5.83 (*p* = 0.006)
**Men**	Urban	9.98 (*p* = 0.01)	1.28 (*p* = 0.01)	−2.28 (*p* = 0.01)	11 (*p* = 0.01)
**Men**	Rural	4.94 (*p* = 0.18)	1 (*p* = 0.05)	−2.2 (*p* = 0.06)	15.6 (*p* = 0.06)

SarQoL = SarQoL questionnaire; SPPB = Short Physical Performance Battery; NRS = Numeric Rating Scale for pain; PPT = Pressure Pain Threshold.

**Table 6:** Control group parameters at initial and final assessments.

Parameters		Mean Value	SD	Min Value	25th Percentile	Median Value	75th Percentile	Max Value
**Total SarQoL**	**T1**	59.79	4.02	51.4	58.15	59.7	61.5	68.7
	**T2**	59.69	3.57	52.8	57.67	59.05	61.86	68.9
**SPPB**	**T1**	7.11	0.74	6	7	7	8	8
	**T2**	7.36	0.59	6	7	7	8	8
**NRS**	**T1**	8.72	9.5	6	7	7	8	8
	**T2**	6.61	0.59	5	6	7	7	8
**PPT**	**T1**	72.16	9.41	53	68	73.5	78	96
	**T2**	72.36	9.05	54	67	73.5	77	94

T1 = initial; T2 = final; SarQoL = SarQoL questionnaire; SPPB = Short Physical Performance Battery; NRS = Numeric Rating Scale for pain; PPT = Pressure Pain Threshold.

**Table 7:** Mean differences between initial and final measurements for SarQoL, SPPB, NRS, and PPT, respectively, by sex and environment in CG (*p*-values from the Wilcoxon signed-rank tests).

Sex	Environment	SarQoL_diff	SPPB_diff	NRS_diff	PPT_diff
**Women**	Urban	0.40 (*p* = 0.40)	0.27 (*p* = 0.08)	−0.45 (*p* = 0.09)	0.36 (*p* = 0.36)
**Women**	Rural	−0.78 (*p* = 0.40)	0.21 (*p* = 0.08)	−0.64 (***p* = 0.01**)	−0.5 (*p* = 0.07)
**Men**	Urban	0.97 (*p* = 0.93)	0.28 (*p* = 0.15)	0 (*p* = 1)	0.57 (*p* = 0.37)
**Men**	Rural	−0.97 (*p* = 0.28)	0.25 (*p* = 0.31)	−1 (*p* = 0.12)	−0.25 (*p* = 0.87)

SarQoL = SarQoL questionnaire; SPPB = Short Physical Performance Battery; NRS = Numeric Rating Scale for pain; PPT = Pressure Pain Threshold.

## Data Availability

Data is unavailable due to privacy.
